# Air Travel Considerations for the Patients With Heart Failure

**DOI:** 10.5812/ircmj.17213

**Published:** 2014-06-05

**Authors:** Morteza Izadi, Mohammad Javad Alemzadeh-Ansari, Davood Kazemisaleh, Maryam Moshkani-Farahani

**Affiliations:** 1 Health Research Center, Baqiyatallah University of Medical Sciences, Tehran, IR Iran; 2 Department of Cardiology, Tehran Heart Center, Tehran University of Medical Sciences, Tehran, IR Iran; 3 Department of Cardiology, Baqiyatallah University of Medical Sciences, Tehran, IR Iran

**Keywords:** Aerospace Medicine, Heart Failure, Stress, Travel, Venous Thromboembolism

## Abstract

**Context:**

Prevalence of patients with heart failure (HF) is increasing in worldwide, and also the number of people with HF traveling long distances is increasing. These patients are more prone to experience problems contributed air travel and needs more attention during flight. However, observational studies about problems of HF patients during flight and appropriated considerations for them are limited.

**Evidence Acquisition:**

We evaluated the conditions that may be encountered in a HF patient and provide the recommendations to prevent the exacerbation of cardiac failure during air travel. For this review article, a comprehensive search was undertaken for the studies that evaluated the complications and considerations of HF patients during flight. Data bases searched were: MEDLINE, EMBASE, Science Direct, and Google Scholar.

**Results:**

HF patients are more prone to experience respiratory distress, anxiety, stress, cardiac decompensation, and venous thromboembolism (VTE) during air travel. Although stable HF patients can tolerate air travel, but those with acute heart failure syndrome should not fly until complete improvement is achieved.

**Conclusions:**

Thus, identifying the HF patients before the flight and providing them proper education about the events that may occur during flight is necessary.

## 1. Context

Approximately two billion passengers undertake international and domestic air travels every year. Today, traveling by airplane has become increasingly common and accessible (1, 2). There are some cardiovascular conditions that air travel could be worsen the patient condition such as decompensate heart failure (HF). Although given the right aircraft, on-board equipment and appropriately qualified and experienced escort personnel, aircraft can act as flying intensive care units and carry extremely ill travelers (3). However our ability to care for patients with cardiovascular diseases improves, an increasing number of people with cardiovascular diseases will be traveling long distances. In the other hand, despite availability of new therapies, the prevalence of HF and hospital admission rates have increased and the World Health Organization has recognized HF as an epidemic condition in this century (4, 5). The 2012 statistics from the American heart association list HF as one of the leading cause of hospitalization and death in United States and Europe (6). Incidence of HF increased from 296 per 100,000 person-years in 2000 to 390 per 100,000 person-years in 2007 (7). Statistics showed that prevalence of HF in US adults (age more than 18 years) in 2010 was about 6.6 million (2.8%) and estimated that this increase in future and reach about 9.6 million people in 2030 (25% increase in prevalence) (6). Despite increasing the prevalence of patients with HF in the world and attention to this point that these patients are more prone to experience problems contributed air travel and requires more attention during flight, but there is little research to guide recommendations for HF patients wishing to travel by plane. Thus, in this study, we tried to evaluate the considerations for prevention of worsening of cardiac failure during flight based on the available evidences.

## 2. Evidence Acquisition

A comprehensive search was undertaken using MEDLINE database for the studies and guidelines that evaluated complications and considerations of HF patients during flight. Our search included the MESH headings heart failure, cardiac failure, congestive heart failure, and heart decompensation. We also searched under the MESH headings travel, aerospace, medicine, and transportation, and the text words flight and flying. We sought additional articles by performing the same search strategy in the databases of EMBASE, Science Direct, and Google Scholar. We then combined all searches and removed the duplicate articles. The total number of potential articles in primary search was 144 studies. We excluded irrelevant articles by reading their title and abstract. Finally, 39 studies were used for this review article. Two of our 4 authors reviewed studies independently and discrepancies were resolved by discussion.

## 3. Results

### 3.1. Effect of Cabin Pressure on Heart Failure Patients

Cabin pressure can affect the health of travelers in many ways, including hyobaric hypoxia affecting those with pre-existing HF, chronic obstructive pulmonary disease, or haematological disorders. Commercial flights usually cruise at altitudes of 7010-12498 meter above sea level; however the passenger cabin is pressurized to an altitude of 1524-2438 m (8-10). Most healthy individuals tolerate this cabin pressure (11). Cabin pressurization to 2438 m lead to reduction in the atmospheric pressure of the cabin. Thus at the maximum cabin altitude of 2438 m, the arterial oxygen partial pressure (PaO2) decrease from 95 mmHg to 60 mmHg (12). In healthy travelers, these pressures lead to a 3-4% decrease in systemic oxy-hemoglobin saturation (9, 13). However, many travelers with pre-existing diseases such as HF, chronic obstructive pulmonary disease, or hematological disorders have a reduced baseline PaO2, so reduced cabin pressure leads to further reduction of oxygen saturation. This condition was worsen with increasing flight times (11, 12). For example, a recent study showed that travelers with HF significantly experience more respiratory distress during flight than other travel (HR: 0.16; 95% CI: 0.30 - 0.97) (14).

Some methods are available to assess the need for oxygen during flight. Hypoxic-challenge test is a good method to assess whether travelers with pre-exciting disorders require oxygen during flight. The maximum cabin altitude of 2438 m can be simulated at sea level with a gas mixture containing 15% oxygen in nitrogen. Individuals breathe the hypoxic gas mixture for 20 min during the test oxygen saturation is monitored. Furthermore, arterial blood gases are measured before and at the end of the test. If PaO2 drops below 50 mmHg, or, if the oxygen saturation drops below 85%, the traveler would need oxygen during flight (11). A recent study showed that patients with stable chronic HF in NYHA functional class II or III on stable treatment could tolerate inspiring 15% oxygen for one hour and no worsening of symptoms were occurred despite reductions in arterial oxygen saturation and increases in mean arterial pressure and systolic pulmonary artery pressure (15). The guidelines from the British Thoracic Society suggest hypoxic-challenge testing in HF patients who are hypoxemic at sea level, especially those with coexistent lung or pulmonary vascular disease (16). The guidelines of Aerospace Medical Association suggest hypoxic-challenge testing as the gold standard for suspicious travelers. These guidelines recommend using oxygen during flight for stable HF travelers with a sea-level PaO2 of 70 mmHg or lower, or with an expected PaO2 of 55 mmHg or lower during flight (17). Cabin pressure also could be affected gas in body cavities. Base on Boyle’s law, the volume that a gas occupies is inversely proportional to the surrounding pressure. Thus, with altitude increase, the low cabin pressures cause to expansion of gas trapped in body cavities up to 30% (10, 11). Against healthy travelers that this expansion can result in minor abdominal cramping or baron-trauma to the ears, the travelers who have undergone recent surgical procedures are at increased risk of problems related to gas expansion such as bowel perforation or wound dehiscence (18, 19). In addition, stretching gastric or intestinal mucosa may result in hemorrhage from ulcer (17). Abdominal bloating is a common complain of patient with right sided-HF (20), and more intestinal gas expansion at altitude could cause additional discomfort in patient with HF. For this reason, it is prudent to avoid gas-producing foods in the days before a scheduled flight or during flight.

### 3.2. Stress of Air Travel

Stress and anxiety is more common in patients with HF (21). About 40% of patients with HF may suffer from major anxiety, and overall anxiety levels are 60% higher than levels seen in healthy elders. The patients with HF, compared with other patients with cardiac disease and patients with cancer or lung disease have similarly high or worse anxiety levels (21, 22). Moreover, anxiety may affect the outcomes of patients with HF. Riedinger et al. documented that anxiety was associated with a higher incidence of adverse cardiac events and cardiac death in the subsequent 6-10 years, among patients with recent acute myocardial infarction and decreased LVEF (23). In another study, De Jong et al. showed that HF patients with high anxiety had a shorter (HR: 2.2; 95%CI: 1.1-4.3) period of event-free survival than patients with lower anxiety (24). In the other hand, anxiety during flight is a common phenomenon, even in healthy travelers. About 40% of travelers experience the anxiety during take-off and landing (25). The patients with HF are more susceptible to increased sympathetic activity with altitude exposure, resulting in hemodynamic changes including vasoconstriction and tachycardia as well as release of inflammatory mediators (26). Increases in sympathetic activity cause to increase in the systemic vascular resistance, blood pressure, and heart rate (27). Also, some studies have shown that patients evacuated by helicopter and fixed-wing aircrafts experienced increased anxiety and heightened levels of catecholamine during flight (28). These changes in patients with HF may cause to cardiac decomposition, especially in those with pre-exciting anxiety disorder. Moreover, preflight activities can cause patients with compromised heart activity to overexert their body; which this may happen because of patients trying to meet time constraints and carrying heavy baggage through check-in or the long walking distance to and from their gate areas (29). Some considerations for control of anxiety during flight was recommended such as providing information about theory of flight and flight safety, and training relaxation techniques including simple breathing techniques, guided visualization, and progressive muscle relaxation (30).

### 3.3. Dehydration During Prolonged Flight

The relative humidity in the cabin gradually falls on high altitude and prolonged flights (31). Low humidity within the cabin of plane coupled with decreased fluid intake causes dehydration contribute to the deleterious effect of air travel on cardiac patients by effectively decline preload and subsequently cardiac output. The noticeable effect of low humidity on travelers such as drying of the skin and mucous membranes are present after 3-4 hours of flight (32). A study showed that low humidity in prolonged air travel can cause to increase in mean plasma osmolarity, mean urine osmolarity and urine specific gravidity, indicating dehydration (33); whereas some studies did not confirmed dehydration secondary to low humidity (34, 35). Moreover, alcohol or coffee drinking (which promotes diuresis), together with the lower humidity of the cabin, may lead to some degree of dehydration (31, 36). Although, most healthy individuals tolerate this dehydration during prolonged flight, but in HF patients especially those receiving diuretics, imbalance of fluid-electrolyte may occurred. Thus, they should be particularly careful about their salt intake and avoid excess consumption of alcohol or coffee.

### 3.4. Patients With Acute Heart Failure Syndrome Who Planning Air Travel

In general, acute heart failure syndromes (AHFS) can be defined as the new onset or recurrence of gradually or rapidly developing symptoms and signs of HF requiring urgent or emergent therapy and resulting in hospitalization. A variety of other overlapping terms have been used in the literature, including Acute HF, acute decompensated HF, and acute decompensation of chronic HF. AHFS is likely to increase in the future, and the high readmission rate of patients with Acute HF is an important issue in the world (37), despite improved rates of in-hospital mortality. The readmission rate of patient with HF is a high; 50% at 6 months (38). An important cause of AHFS is an acute coronary syndrome. However, improvements in the management of patients with acute coronary syndrome were associated with significant reductions in the rates of new-onset HF and mortality (39). The VALIANT study assessed the incidence of and prognostic factors for HF hospitalization among survivors of high-risk acute myocardial infarction. In this study, 1489 patients who died or experienced a non-fatal cardiovascular event (including HF) within the first 45 days and 2174 patients with prior history of HF were excluded. This large study showed that of remain 11040 stable post-myocardial infarction patients, 1139 (10.3%) developed new-onset HF during the median 25-month follow-up; and the most important predictors of HF were older age, antecedent diabetes, prior MI before index MI, and reduced renal function (40). The British Cardiovascular Society divided the travelers who have suffered an acute coronary syndrome based on LVEF into three groups: very low risk, medium risk, and high risk. Very low risk group include the patients with EF more than 45%. Also they had age below than 65 years with the first event, successful reperfusion, no other complications, and no planned investigations or interventions. Medium risk group included patients with EF more than 40% with no symptoms of HF, no evidence of inducible ischemia or arrhythmia, and no further investigations nor interventions planned. High risk group referred to patients with EF less than 40% with signs and symptoms of AHFS. Also these patients wait to further investigation with a view to re-vascularisation or device therapy. This report recommended safely flights as early as 3 days after the event for very low risk group, flights from 10 days onwards for medium group. But, those at high risk or awaiting further investigation/treatment, flying should be deferred until a more stable situation is achieved (3). Except acute coronary syndrome, episodes of AHFS may be provoked by anemia or infection on the background of chronic HF. When the patients with AHFS are identified and treated, most cases should be stabilized within 6 weeks and should be safe to fly (3). Regardless of etiology of AHFS, other references emphasis that the decompensated HF is one of the contraindication of air travel (16, 17, 41, 42). The British Thoracic Society also confirmed above data and recommended that if air travel cannot be avoided for the patients with decompensated HF, they should have oxygen during flight (16). However in this condition, right aircraft, on-board equipment and appropriately qualified and experienced escort personnel should be provided (3).

### 3.5. Patients With Stable Chronic Heart Failure Who Planning Air Travel

Travelers with stable chronic HF without recent changes in symptoms or medication are likely to be able to tolerate the mild hypoxia of the aircraft cabin environment even if they have very low LVEF (14, 15, 43). Ingle et al. in a study conducted a survey in a representative cohort of ambulatory patients with stable, well managed chronic HF to discover their experiences of air travel. Results showed that 65% of patients with HF who travelled by air experienced no health-related problems, although 35% of them experience health problems such as breathlessness, dizziness, swollen ankles, headache, and chest pain, mainly at the final destination (14). The British Thoracic Society recommended that patients with stable HF who are hypoxemic at sea level with coexistent lung or pulmonary vascular disease should be considered for Hypoxic-challenge test. Also they suggested that if patients with stable HF in NYHA functional class I-III (without significant pulmonary hypertension) can fly without oxygen (16). However the Aerospace Medical Association advised in-flight medical oxygen for stable patients with NYHA functional class III-IV HF or baseline PaO2 less than 70 mmHg (17). The British Cardiovascular Society regarded the time of flight and suggested that, for patients with stable HF including NYHA functional class III and IV, short-term (up to 1 hour) hypoxia at rest produces no significant worsening effects; and periods of up to 7 hours are tolerated by those with mild to moderate stable HF (NYHA functional class II) (3).

### 3.6. Association of Venous Thromboembolism and Heart Failure During Air Travel

Chronic HF has long been proposed as a risk factor for venous thromboembolism (VTE) (44). This association relates to abnormal and slow flow patterns in dilated, poorly contracting cardiac chambers, which causes to venous stasis resulting mural thrombi. This may predispose to the development of VTE as well as peripheral arterial emboli. Thus, patients with worsening left ventricular systolic function are more prone to risk of thromboembolic events. Howell et al. indicated HF is an independent risk factor for VTE, and the risk increases markedly as the EF decreases. They found that a LVEF between 20% and 44% was associated with an increased risk of VTE, with an odds ratio of 2.8 (95% CI 1.4–5.7%); and LVEF less than 20% was associated with an odds ratio of 38.3 (95%CI 9.6–152.5%) for VTE (45). Dries et al. showed that the overall annual incidence of thromboembolic events were more in women with HF (2.4% in women vs. 1.8% in men). Also on multivariate analysis, they observed that a decline in LVEF independently associated with thromboembolic risk in women (relative risk per 10% decrease in LVEF 1.53, 95% CI 1.06-2.20), but no relation was observed in men (46). In the other hand, a direct relation between VTE and long-distance air travels has been documented in previous studies. Kuipers et al. in a systematic review showed that the long-distance travel increased the risk of VTE approximately 2 to 4-fold (47). Chandra et al. in a meta-analysis found that travel is associated with a 3-fold higher risk for VTE, with a dose-response relationship of 18% higher risk for each 2-hour increase in travel duration (48). The WRIGHT project in phase 1 reported that long-distance air travel (more than 4 hours) approximately doubled the risk of VTE. The absolute risk of VTE per more than 4 hour flying, in healthy individuals, is 1 in 6000, rising to about 1 to 1000 travelers for frequent flights (were taken in the four–week exposure period) and longer journeys (49). According to above data, it can be concluded that patients with HF who are planning for air travel are at higher risk of VTE. Based on current guidelines, there are general recommendations for preventing VTE during flight including perform regular leg exercises (e.g. ankle movements, isometric exercises, and walking), avoid excessive alcohol consumption, and avoid the use of tranquillizers and sleeping pills whilst sitting position (50-53). The Aerospace Medical Association considered the patient with uncontrolled HF as a moderate risk. Another study divided travelers with HF base on their LVEF. The patients with LVEF between 20% and 44% was considered as a moderate risk group for VTE and those with LVEF less than 20% was categorized as a high risk group. This study was recommended compression stockings, mobilization, hydration, and aisle seating for moderate risk group; and additional one injection of low-molecular-weight heparin prior to flight in those not currently treated with warfarin for high risk group (41).

### 3.7. Other Considerations for Heart Failure Patients Who Planning Air Travel

It was recommended that all patients with HF should first consult their doctors before traveling, and if they are to attempt a prolonged air travel, must be able to walk 100 yards and climb 12 steps (54). The HF patients may also be more prone to the symptoms of altitude sickness such as shortness of breath and profound fatigue (55). Patients with HF must carry a list of their medications using the generic name and dosages for each drug. It would be better that they bring extra medications and store them in carry-on luggage for the flight. A baseline electrocardiogram and the name and address of the patient’s treating physician should be carried. Furthermore, it will be very helpful to the evaluating physician that the patient carry a brief letter from the patient’s physician describing the traveler’s medical problems. Patients with cardiac pacemaker or implantable cardioverter defibrillator could be safely fly and will not be affected by airline metal detectors (56); although in rare cases, these devises may set off airport metal detectors (41). These patients should carry a copy of wallet card identifying type of pacemaker or implantable cardioverter defibrillator and those with pacemaker should carry a copy of their electrocardiogram with and without pacing (41, 55, 57).

### 3.8. Study Limitations

This review article has some limitations. First, we just searched English articles published in the selected databases and did not search for articles in other languages. Second, we used limited internet databanks and just used references which were available in full text.

## 4. Conclusions

The patients with HF are more prone to experience problems contributed air travel and needs more attention during flight. Altitude and cabin pressure causes to hyobaric hypoxia resulting reduced arterial oxygen partial pressure and more respiratory distress in HF patients. The hypoxic-challenge testing was suggested for HF patients who are hypoxamic at sea level, especially those with coexistent lung or pulmonary vascular disease. Also, in-flight oxygen for stable HF travelers with a sea-level PaO2 of 70 mmHg or lower, or with an expected PaO2 of 55 mmHg or lower during flight was recommended. The HF patients are more susceptible to experience anxiety during flight resulting in hemodynamic changes including vasoconstriction, tachycardia, and subsequently decompensated HF. Thus, training relaxation techniques are more necessary to these travelers. Attention to salt intake and avoid excess consumption of alcohol or coffee in HF patients in order to prevention of dehydration is essential. Although stable HF patients, even in NYHA functional class III or IV, can tolerate air travel, but those in the stage of AHFS should not fly until complete improvement is achieved. Patients with HF are prone to experience VTE following prolonged air travel, especially those with lower LVEF. Also, patients with HF should first consult their doctors before traveling, and carry a list of their medications, extra medications and store them in carry-on luggage, a baseline electrocardiogram, and the name and address of the own physician. Thus, identifying these patients before flight and proper education to them about the events that may occur during flight is necessary. Also physicians at the airport and crew in plane should be aware about the problems that may occur for these patients, to take the necessary action at the right time. This review article evaluated the complications of HF patients and also reviewed the considerations to prevent the exacerbation of cardiac failure during flight. However, to achieve better results, further investigations, especially randomized clinical trials studies are needed.
